# Augmented inferior rectus transposition with medial rectus recession in treatment of chronic unilateral sixth nerve palsy

**DOI:** 10.1186/s12886-022-02552-2

**Published:** 2022-08-08

**Authors:** Mohamed F. Farid, Ahmed A. Khater, Ahmed M. Elbarky

**Affiliations:** 1 Ophthalmology Department, Benha University, PO Box: 13511, 1 Fareed Nada Street, Benha, Egypt; 2 Sheikh Khalifa Medical City (SKMC), Abu Dhabi, UAE

**Keywords:** Sixth nerve palsy, Rectus muscle transposition, Inferior rectus muscle

## Abstract

**Background:**

to report the results of augmented inferior rectus muscle transposition (IRT) in management of chronic sixth nerve palsy.

**Methods:**

a retrospective review of medical records of patients with chronic complete sixth nerve palsy who were treated by augmented full thickness IRT to the lateral border of the paralyzed lateral rectus muscle. Patients were selected for IRT if there was more limitation of abduction in inferior gaze associated with V- pattern esotropia. Medial rectus recession (MRRc) was performed in case of positive intraoperative forced duction. Effect on primary position esotropia, face turn, amount of V-pattern and limitation of ocular ductions were reported and analyzed.

**Results:**

the review revealed 11 patients (7 males) with chronic unilateral sixth nerve palsy who were treated by simultaneous augmented IRT and MRRc. Causes of sixth nerve palsy were trauma (6 cases), vascular (3 cases), inflammation and congenital (one case each). Mean age of the patients at the time of surgery was 35.6 years (range; 11–63) and mean follow up was 8.6 months (range; 6–13). Postoperatively, average correction of esotropia, V-pattern, face turn and limited abduction were 35.9 PD, 11.4 PD, 25.9° and 2.2 unit, respectively (*p* < .00). Postoperative complications in the form anterior segment ischemia, symptomatic induced vertical deviations were not found.

**Conclusions:**

In cases of chronic unilateral sixth nerve palsy associated with more limitation of abduction in downgaze and V-pattern esotropia, augmented IRT could be considered as an effective and safe modality.

## Introduction

Various forms of vertical rectus muscle transposition (VRT) have been proposed to overcome esotropia, face turn and limited abduction associated with complete chronic sixth nerve palsy. They include partial thickness VRT [1], full thickness VRT [2] and isolated superior rectus muscle transposition (SRT) [3, 4]. Enhancement of the effect of each transposition technique could also be obtained by application of certain augmentation techniques which include scleral fixation [5], myopexy sutures [6] or combination of both [7].

Isolated SRT, as a form of single vertical rectus muscle transposition was aiming at reducing the incidence of anterior segment ischemia especially when combined with medial rectus muscle recession, and it was found effective in improving limited abduction and esotropia in chronic abducens palsy [8–10]. Recently, inferior rectus muscle transposition (IRT) as another modality of single vertical rectus muscle transposition in treatment of chronic sixth nerve palsy was recently introduced [11]. The aim of the current study is to evaluate the results of augmented IRT in treatment of chronic abducens palsy.

## Patients and methods

This study was approved by institutional review board of Benha University hospital and it adhered the tenets of the Declaration of Helsinki. The medical records of consecutive patients with chronic non resolving complete sixth nerve palsy who underwent augmented IRT with a minimum of 6 months follow up were retrospectively reviewed. Patients were included if their sixth nerve palsy was more than 6 months duration. Patients were selected for IRT surgery if limited abduction was significantly more pronounced in the inferior gaze with associated V-pattern esotropia.

Data acquisition include patients’ demographics in the form of age at time of surgery, duration of follow up, gender, etiology and duration of sixth nerve palsy and presence of diplopia at the primary position. Full comprehensive ophthalmic and orthoptic examinations were performed preoperatively and at each postoperative visit including corrected visual acuity, cycloplegic refraction and fundus examination. Esotropia in the forced primary position was measured at both distant and near fixation using alternate prism cover test with values of distant esotropia were used for evaluation. The degree of esotropia was measured in up and downgaze, and V-pattern was diagnosed if the difference between them was ≥15 prism diopter (PD). The degree of face turn was measured in the habitual head position during distant fixation using orthoptic goniometer with 5-degree scale. Degree of limitation of ocular duction was assessed and documented using a 6- point scale [12]. Ocular torsion was objectively assessed by measuring the degree of fundus torsion or the position of the fovea relative to optic disc. Complete sixth nerve palsy was diagnosed by very minimal to no abduction force on active generation test with absent or floating saccades.

Under general anesthesia, the degree of tightness of the medial rectus muscle was assessed at the beginning of surgery using forced duction test, and in case of significant contracture, the medial rectus muscle was recessed in the usual manner by an amount sufficient to relieve the contracture, as evident by negative forced duction test. Through limbal conjunctival incision, the inferior rectus muscle was hooked and cleared from all surrounding Tennon’s capsule and fascia. Care was taken to lyse any attachment between the inferior rectus muscle and inferior lid retractors to avoid postoperative lower lid retraction. The inferior rectus muscle was imbricated close to its insertion using double armed 6/0 polyglactin 910 sutures before it was disinserted. The detached muscle was then laterally transposed and was sutured to the sclera in a crossed sword fashion adjacent to the insertion of the paralyzed lateral rectus muscle along the spiral of Tillaux. The transposition was then augmented as described by Farid by passing the needle of a non-absorbable 5/0 polyester suture through the temporal half of the transposed inferior rectus muscle then through the sclera close to the inferior border of the lateral rectus muscle and finally through the inferior third of the lateral rectus muscle 8–10 mm posterior to lateral rectus insertion [5]. Finally, the conjunctiva was closed by interrupted 7/0 polyglactin 910 sutures.

Careful slit lamp examination was performed at each visit to detect any sign of anterior segment ischemia. Values of the last postoperative visit were used for statistical analysis and comparison using paired *t*-test with a *p* value less than 0.05 was considered significant.

## Results

The review reveals 11 patients who underwent unilateral IRT for treatment of unilateral chronic complete sixth nerve palsy. Mean age of patients at the time of surgery was 35.6 (range; 11–63 years) and average amount of follow up was 8.6 months (range; 6–13 months). Out of the 11 patients, 7 patients were males and the left eye was affected in 6 patients. Trauma was the leading cause of sixth nerve palsy (6 cases) followed by vascular causes (3 cases) then inflammatory and congenital (one cases each). Diplopia at the primary position was present in 9 patients at presentation and completely disappeared in all patients after surgery. Data of fundus torsion before and after surgery was available for only 4 patients. In those patients, no induced torsion was detected after surgery. Intraoperatively, medial rectus contracture was encountered in all cases as evident by positive forced duction test which mandated simultaneous medial rectus muscle recession by an amount sufficient to relieve the contracture (mean 4.5, range; 3.5–5.5 mm). Table 1 shows detailed patients characteristics pre and postoperatively.

**Table 1:** Patients’ demographics and surgical results

								Preoperative						Postoperative				
Patient	Age (yr)	Gender	Side	Etiology	FU (mo)	MRc (mm)	Align	V-pattern	AHP	Abd	Add	Diplopia	Align	V-pattern	AHP	Abd	Add	Diplopia
1	17	M	OS	traumatic	8	4.5	35ET 8HT	25	20°	-5	0	Y	4XT 2HT	15	0°	−2	0	N
2	12	M	OS	SST	6	5	45ET 4HT	25	40°	−5	0	N/A	12ET	10	10°	−3	0	N/A
3	34	M	OD	traumatic	11	4	40ET 6HT	20	35°	−4	1	Y	10ET 2HT	10	5°	−2	0	N
4	52	F	OD	traumatic	9	4	35ET	15	25°	−4	0	Y	6ET	6	0	−2	−1	N
5	23	M	OS	traumatic	7	4.5	45ET 4HT	20	30°	−4	0	Y	15ET 2HT	10	10°	−1	−1	N
6	47	F	OD	traumatic	13	5.5	65ET 4HT	25	45°	−6	1	Y	20ET 4HT	6	20°	−3	0	N
7	11	M	OS	congenital	11	4	35ET 6HT	20	35°	−4	0	N	5ET 4HT	10	0	−3	−1	N
8	36	M	OD	inflammatory	6	3.5	25ET 4HT	15	15°	−4	0	Y	8XT 2HOT	4	0	−2	0	N
9	63	F	OS	CVA	6	4	30ET	20	25°	−4	0	Y	10ET 2HOT	10	5°	−2	0	N
10	58	F	OS	CVA	8	5.5	60ET 4HT	25	45°	−5	1	Y	15ET 2HT	15	10°	−3	0	N
11	39	M	OD	traumatic	10	5	50ET	20	40°	−5	0	Y	5ET	8	10°	−3	−1	Y

*yr* years, *M* male, *F* female, *mo* months, *SST* sagittal sinus thrombosis, *CVA* cerebrovascular accident, *FU* follow up duration in months, *MRc* medial rectus recession, *Align* alignment, *ET* esotropia, *HT* hypertropia, *XT* exotropia, *HOT* hypotropia, *AHP* abnormal head posture, *Abd* abduction, Add’ adduction, *N/A* not applicable

**FIG. 1:**
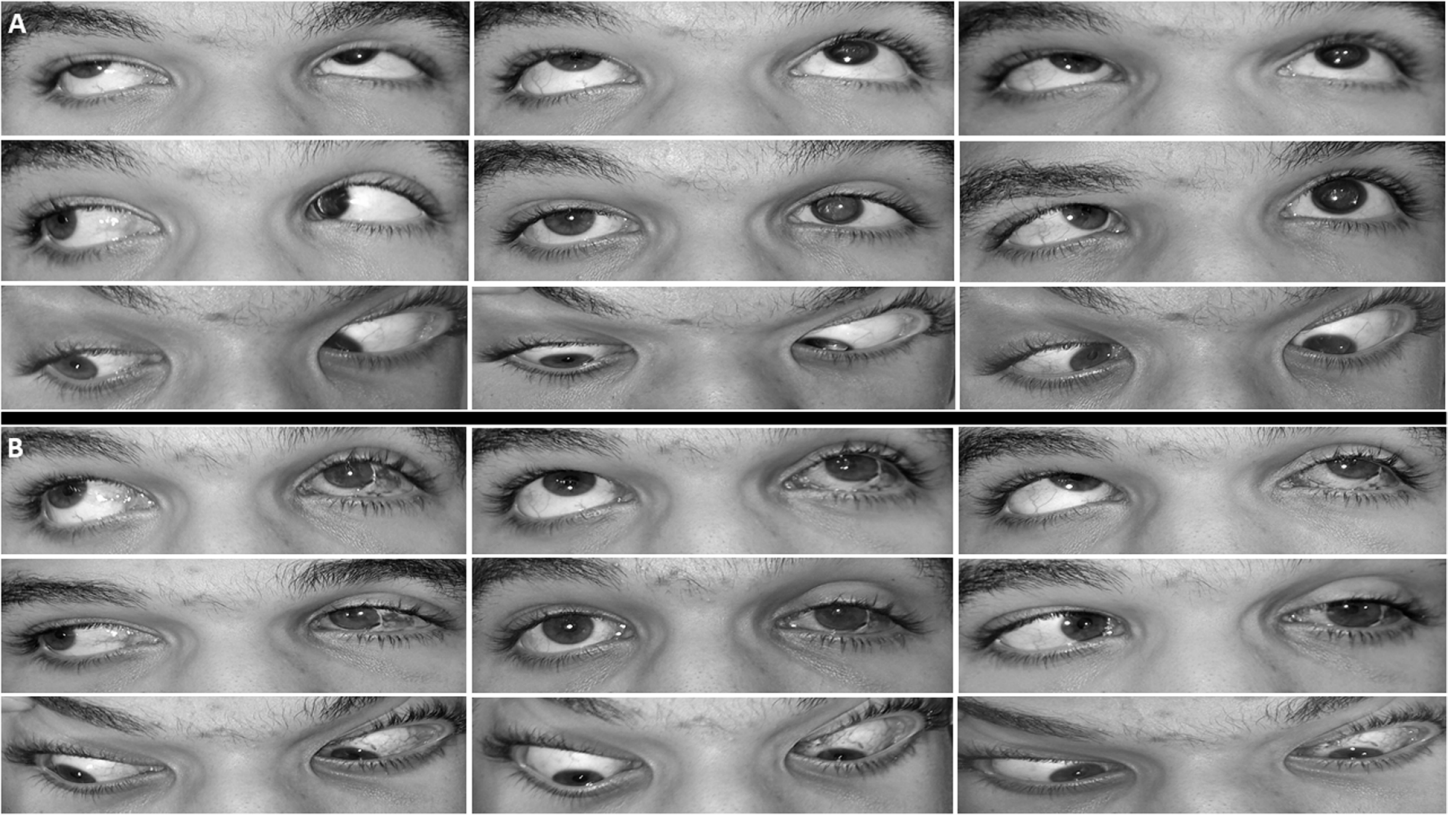
9-gaze clinical photos of a patient with chronic posttraumatic left sixth neve palsy. **A** preoperative photos show 35PD primary position ET which increased in the down compared to the up gaze (V-pattern ET). Limitation of abduction in the left eye was − 5 which was less evident in the upper lateral gaze compared to lower lateral gaze. **B** postoperative photos following left dual augmented IRT combined with 4.5 mm medial rectus recession. Primary position alignment improved to 4PD exotropia and limited abduction to − 2. with collapse of V-pattern. Note; the left pupil was pharmacologically dilated

After augmented IRT, esotropia improved from 42.2 prism diopter (PD) (range; 25 to 60 PD) before surgery to 7.8 PD (range; − 8 to 20 PD) (*p* < .00001). Postoperative small angle consecutive exotropia developed in two patients. However, the exotropia was asymptomatic and of small magnitude (4 and 8PD) so that no further actions were needed. Face turn improved from 32.2° (range; 15 to 45°) to 6.3° (range; 0 to 20°) (*p* < .00001). Limitation of abduction of the affected eye improved from − 4.5 unit (range; − 4 to − 5 unit) to − 2.3 unit (range; − 1 to − 3 unit) (*p* < .00001), while adduction declined from 0.2 unit (range; 0 to1 unit) to − 0.3 unit (range; − 1 to 0 unit) (*p* = 0.0018). Postoperatively, minimal (− 1 unit) induced limitation of adduction was recorded in 3 cases (Fig. 1, Table 2).

**Table 2:** Main parameters of the study before and after surgery

Item (mean ± SD)	Preoperative (range)	Postoperative (range)	Improvement (range)	***p***-value
**Esotropia (PD)**	42.2 ± 12.3 (25 to 60)	7.8 ± 8.2 (−8 to 20)	35.9 ± 7.3 (25 to 50)	< .00001
**V-pattern** **(PD)**	20.9 ± 3.7 (15 to 25)	9.4 ± 3.4 (4 to 15)	11.4 ± 2.9 (9 to 19)	< .00001
**Face turn** **(degree)**	32.2 ± 10 (15 to 45)	6.3 ± 6.3 (0 to 20)	25.9 ± 6.6 (15 to 35)	< .00001
**Abduction (unit)**	−4.5 ± 0.6 (− 4 to −5)	−2.3 ± 0.6 (−1 to −3)	2.1 ± 0.6 (1 to 3)	< .00001
**Adduction (unit)**	0.2 ± 0.4 (0 to1)	−0.3 ± 0.5 (−1 to 0)	− 0.5 ± 0.4 (− 1 to 0)	0.0018

The average amount of V-pattern (difference between deviation in downgaze and upgaze) collapsed from 20.9 PD (range; 15 to 25 PD) preoperatively to 9.4 PD postoperatively (range; 4 to 15 PD) (*p* < .00001) (Fig. 1). Out of 8 patients with preoperative HT, 6 patients had reductions of up to 6 PD, with one showing a reversal to a post-operative hypotropia. One patient with no pre-existing vertical deviation developed a hypotropia. In both patients who developed postoperative hypotropia., It was asymptomatic and no further actions were required. After surgery, esotropia and face turn improved by means of 35.9PD (range; 25 − 50PD) and 25.9 degrees (range; 15–35°) respectively. Average improvement of V-pattern was 11.4 PD (range; 9–19 PD) Average improvement of limited abduction was 2.1 (range; 1 to 3) while average decline of adduction was − 0.5 (range; − 1 to 0) (Table 2).

Mild induced vertical deviation in the form induced hypotropia of 2 PD was reported in two patients. Mild asymptomatic induced limitation of adduction was reported in two cases. No other postoperative complications in the form of anterior segment ischemia or induced torsional diplopia were reported in any patient.

## Discussion

Transposition of vertical rectus muscles has been considered as mainstay of treatment of complete sixth nerve palsy. Despite their various forms, all transposition procedures aim for improvement of the compromised abduction with correction of esotropia, face turn and expansion of the field of binocular single vision [13]. Traditional VRT procedures involve the simultaneous lateral transposition of part or the whole of both vertical rectus muscles at the same time. Single vertical rectus muscle transposition in the form of superior rectus transposition was proposed aiming for reduction of the incidence of postoperative anterior segment ischemia, especially when medial rectus muscle contracture mandates simultaneous recession of the medial rectus muscle [3, 4]. Since then, SRT has been studied in many articles concerning cases with defective ocular abduction secondary to chronic sixth nerve palsy and esotropic Duane retraction syndrome and its efficacy has been well established [4, 8, 9] [8–10]. Recently, a large retrospective analysis of induced vertical deviations secondary to SRT in sixth nerve palsy and esotropic Duane retraction syndrome was performed. The study comprised 69 patients who underwent SRT, 32 patients with sixth nerve palsy and 37 patients with esotropic Duane retraction. The study revealed that 14 patients developed hypertropic shift while 23 patients developed hypotropic shift. Out of these cases, 5 cases developed persistent vertical diplopia which required intervention [10].

The idea of IRT was first proposed by Velez and colleagues in 2017 to treat complete sixth nerve palsy in a series of 5 patients [11]. Because of their experience with induced hypertropia after SRT in sixth nerve palsy [9], they started transposing the inferior rectus muscle in patients with preoperative hypertropia. Their case series comprised 5 consecutive patients with sixth nerve palsy associated with preoperative hypertropia or more defective abduction in the down gaze. In terms of results, IRT scored significant improvement of esotropia, face turn and limited abduction with no reported cases of symptomatic induced vertical deviation. In the current study, we have evaluated our results with IRT on a series of 11 patients with chronic sixth nerve palsy. In general, we have found that IRT produces significant correction of esotropia, face turn, limited abduction with no reported cases of symptomatic induced vertical deviation, torsional diplopia or anterior segment ischemia.

In Velez study [11], average esotropia improvement was 27 PD, face turn correction was 26.4° with an average improvement of limited abduction by 1.0 unit. Compared with Velez study, our results are quite superior with an average improvement of esotropia by 35.9 PD, face turn correction by 25.9° and an average improvement of abduction limitation by 2.2 unit. We believe that our technique of dual augmentation of transposition compared with single muscle to muscle augmentation technique used by Velez and colleagues could explain such discrepancy. In addition, smaller number of patients in Velez study (5 patients) compared with the current one (11 patients) could also plays a role in such difference. One of the similar aspects between the current study and that of Velez is that IRT in both studies lead to collapse of the preoperative hypertropia by an average of 2.5 PD in the former and 1.2 PD in the latter. In Velez study, one patient developed 2PD induced hypertropia while another patient developed 3PD induced hypotropia. In the current study, two patients developed 2 PD induced hypotropia. In both studies, the induced vertical deviations were innocuous and of small magnitude which warranted no further interventions.

Recently, Sener and colleagues in 2019 evaluated IRT in management of 7 esotropic Duane retraction syndrome patients who had either more defective abduction in the inferior gaze or had V-pattern esotropia [14]. In their study, average esotropic correction was 19.6 PD, V-pattern collapse was 19.9 PD, face turn improvement was 16.4° with an average improvement of limitation of abduction by 1.3 unit. Induced hypertropia was reported in two patients which mandated secondary surgical intervention in one patient. Comparatively, the results of the current study in correction of esotropia, face turn and limited abduction are slightly better than those of Sener study. However, we believe that comparisons are quite irrelevant because of different characters of patients in both studies (esotropic Dune retraction syndrome vs. sixth nerve palsy). In addition, different augmentation techniques were used in Sener study which vary between resection of inferior rectus muscle in some cases and posterior scleral fixation suture in other cases. Nevertheless, we believe that Sener study holds a beneficial role in the evaluation of the efficacy and safety pattern of IRT procedure.

Limitations of the current study include its retrospective nature, small number of included patients, in addition to paucity of data regarding the effect of IRT on fundus torsion. However, and taking into account the relative recentness of IRT procedure, this study provides valuable insights into outcomes of a recently practiced transposition procedure. We have found that IRT is effective in management of esotropia, face turn, limited abduction associated with chronic sixth nerve palsy with collapse of preexisting V-pattern. In addition, the impact of IRT on collapse of preexisting hypertropia with low incidence of significant induced hypotropia, which was observed in Velez study as well as in the current study, could open new insights into potential implications of IRT in cases of Sixth nerve palsy with preoperative hypertropia. Base on its efficacy and low rate of significant postoperative complications such as induced vertical deviations and torsional diplopia, we believe that IRT could be considered in management options of chronic sixth nerve palsy especially in cases with V-Pattern esotropia and more abduction limitation in the downgaze. However, and as data on outcomes of IRT on cases of sixth nerve palsy which do not exhibit the aforementioned criteria are currently lacking, future studies with larger sample size are recommended.

## Data Availability

The datasets used and/or analysed during the current study are available from the corresponding author on reasonable request.

## Abbreviations

IRT: Inferior Rectus Transposition
MRRc: Medial Rectus recession
VRT: Vertical Rectus Transposition
SRT: Superior Rectus Transposition
PD: Prism Diopters
